# Eating disorders risk and body shape concerns among medical students in Jordan: a cross-sectional study

**DOI:** 10.3389/fpsyg.2026.1816029

**Published:** 2026-06-03

**Authors:** Hana Taha, Areej Saadeh, Suhib Awamleh, Abdel Rahman AlRamahi, Sara A. Haddadin, Omar Almuhaisen, Farah Almasad, Laila Slihat, Zaid Altawil, Amira Masri, Linus Jönsson

**Affiliations:** 1 Department of Family and Community Medicine, School of Medicine, The University of Jordan, Amman, Jordan; 2 Department for Neurobiology, Care Sciences and Society, Karolinska Institutet, Solna, Sweden; 3 Department of Pediatrics, Division of Child Neurology, School of Medicine, The University of Jordan, Amman, Jordan

**Keywords:** body mass index, body shape questionnaire, eating attitudes test, Jordan, medical students, psychiatric history

## Abstract

**Introduction:**

Eating disorders are serious, multi-systemic and chronic disturbances in eating behavior among young people. Body image dissatisfaction is a known risk for developing eating disorders; therefore, this study aims to explore the proportion of participants screening-positive for elevated eating disorder risk among medical students in Jordan and their relationship with associated factors including body shape concerns.

**Methods:**

A cross-sectional study that used an online survey to collect the data for 402 undergraduate medical students (117 males; 285 females) from six public universities in Jordan. The surveying tool included a sociodemographic section, Eating Attitudes Test (EAT-26), and Body Shape Questionnaire (BSQ-16B). IBM SPSS Statistics (version 27) was used for descriptive statistics, multivariate analysis and logistic regression to identify the factors associated with the increased risk of eating disorders.

**Results:**

Female students had higher mean EAT-26 scores compared to males (*p* = 0.03), while BSQ-16B scores did not differ by gender. BMI was significantly associated with both eating attitudes and body shape concerns, with obese participants demonstrating higher EAT-26 scores than those with normal BMI (*p* = 0.029) and a graded increase in BSQ-16B scores across the BMI categories (all *p* < 0.001). Students living alone reported higher BSQ-16B scores compared to those living with their families (*p* = 0.029). Participants with a history of psychiatric illness, use of diet pills or laxatives, or recent weight loss greater than 10 kg had significantly higher EAT-26 and BSQ-16B scores (all *p* ≤ 0.047). Females were more likely to fall into the high-risk group compared to males (36.1% vs. 29.9%, *p* = 0.03).

**Conclusion:**

This study revealed that a considerable proportion of medical students in Jordan were screening-positive for elevated eating disorder risk. The strongest associated factors were female gender, higher BMI, rapid weight loss, use of diet pills or laxatives, and a history of psychiatric illness, with a strong correlation observed between eating-disorder risk and body shape concerns. These findings highlight the need for further research and targeted preventive strategies to support the mental and physical wellness of medical students.

## Introduction

Eating disorders (ED) are conditions that involve severe and chronic disturbances in eating behaviors, which may affect physical, psychological, and social function, often involving body image issues in terms of weight and shape as defined by the American Psychiatric Association (American Psychiatric Association, 2025). They are multi-systemic illnesses that involve pulmonary, cardiac, and reproductive systems, as well as other systemic manifestations (Mehler and Rylander, 2015). Common symptoms include self-induced vomiting, misuse of laxatives, restrictive eating, and binge eating (American Psychiatric Association, 2025; Ghamri et al., 2022). Globally, the lifetime risk of ED is 8.4% in women and 2.2% in men based on a systematic review that involved 94 studies conducted between 2010 and 2018 (Galmiche et al., 2019). However, the proportion of individuals at elevated risk of eating disorders is markedly higher among medical students, with a 10.4% risk (Jahrami et al., 2019)These findings are supported by other studies, which also demonstrate a significant difference (Ghamri et al., 2022; Bizri et al., 2020; Motorga et al., 2024). For example, in the Middle East, a study conducted at King Abdul-Aziz University in Jeddah reported that 32.1% of medical students were at elevated risk (Ghamri et al., 2022). These findings are alarming, given the high rate of psychiatric disorders as well as burnout among medical students, showing the need to address ED among this population (Dyrbye et al., 2006; Talih et al., 2018).

Body image dissatisfaction (BID) is common among medical students, as a recent study has shown a prevalence of 34.7% of BID (Torres et al., 2018), and it is known that it is a core risk factor for the development of ED (Kiefer et al., 2000; Power et al., 2024). Among medical students, many risk factors have been identified. Several studies have noted that female gender seems to significantly increase the risk of developing ED and are generally more frequently dissatisfied with their bodies (Ghamri et al., 2022; Kiefer et al., 2000; Belhaj Salem et al., 2025). Likewise, many studies have highlighted that being overweight appears to elevate the risk of developing ED within this population (Ghamri et al., 2022; Motorga et al., 2024; Kiefer et al., 2000). A study conducted at the University of Medicine in Romania noted that other risk factors, such as experiencing sleeping difficulties, feelings of ridicule, family and media pressure, prior ED history, negative events, and family history of mental illness, all seem to increase the risk of the development of ED (Motorga et al., 2024). Other studies confirm these associations (Belhaj Salem et al., 2025; Martins-de-Passos et al., 2024). A wide range of studies on ED and BID have been conducted globally, including some from the Middle East region. However, research on this topic in Jordan remains limited, particularly among medical students. Lifestyle changes and the growing influence of social media create additional pressures on Jordanian youth, with a recent study suggesting an association between social media and BID among Jordanian university students (Shihadeh et al., 2025). A study on disordered eating attitudes among Jordanian adolescents highlighted that socio-cultural factors including Westernization, media influence, and cultural norms, greatly influence ED behaviors (Ghraiybah, 2018), indicating that findings from other countries cannot be simply generalized to Jordan. In line with this, a study at the University of Jordan reported that 14.2% of students were at risk of developing an ED, with students from urban areas being at a higher risk than those from rural areas (Basma Fouad Kilani, 2017). Based on current literature, there is no research that explicitly focused on ED and its relationship with body shape concerns among medical students in Jordan. Therefore, this is the first study in Jordan that aims to investigate the proportion of participant’s screening-positive for elevated eating disorder risk, its relationship with body shape concerns, and identify the associated factors among medical students.

## Methods

### Study design and setting

This cross-sectional study was conducted in Jordan from April 2025 to August 2025. Jordan, according to World Bank Data, is considered a lower-middle-income country in the Middle East (World Bank, 2025). The survey was distributed to all six public universities in Jordan with medical schools, including The University of Jordan, Jordan University of Science and Technology, Hashemite University, Yarmouk University, Mu’tah University, and Al-Balqa’ Applied University.

### Sample size and data collection

The study included 402 undergraduate medical students (117 males; 285 females) across public universities in Jordan. The inclusion criteria included undergraduate medical students from years 1 to 6 who were 18 or older. Any student who did not provide informed consent was excluded. Convenience sampling was used to recruit students due to its feasibility and cost-effectiveness. The data was collected using an anonymous, self-administered online questionnaire that was distributed via social media and online groups dedicated only to medical students. Moreover, the survey was available in Arabic and English to ensure accessibility to all participants. The sample size was considered adequate based on commonly used estimates for cross sectional studies. A minimum of approximately 385 participants is typically required to estimate a proportion with a 95% confidence level and a 5% margin of error, assuming a prevalence of 50%. The final sample size of 402 participants exceeded this threshold. Given the large source population, no finite population correction was applied.

### Measurement tool

The survey included three main sections. First was the demographic and socioeconomic section, which collected information such as gender, age, university, academic year, GPA, income level (in JOD), place of residence, living arrangements, governorate, marital status, parents’ occupation, parents’ education, and number of family members. In addition to that, mental health history was collected, such as family history of mental health disorder, previous psychiatric diagnosis, and whether they received any treatment. Some Eating-related behaviors, such as the use of diet/laxative pills and significant weight loss, were also assessed in the survey. Regarding Body mass index (BMI), it was calculated as weight (in kg) divided by height (in m^2^), both of which were provided by the participants themselves. The Second section included the Eating Attitudes Test (EAT-26), a 26-item questionnaire presented in both Arabic and English. The responses were rated on a 6-point scale, from “always” (Bizri et al., 2020) to “never” (American Psychiatric Association, 2025), with total scoring points ranging from 0 to 78. A score above or equal to 20 indicates a high risk (Garner et al., 1982). It is important to note that the EAT-26 is a validated screening tool used to identify individuals with elevated risk or symptoms suggestive of disordered eating, and it does not provide a clinical diagnosis of eating disorders. The Third section assessed body shape concerns using the Body Shape Questionnaire (BSQ-16B). This 16-item questionnaire also rates responses on a 6-point scale, from “always” (Bizri et al., 2020) to “never” (American Psychiatric Association, 2025), with a total scoring range of 16 to 96. A higher score is indicating a greater concern about body shape (Al-Subaie et al., 1996). It was also available in both Arabic and English. The Eating Attitudes Test (EAT-26) is a widely used and validated screening tool for assessing disordered eating attitudes. It has demonstrated good internal consistency, with Cronbach’s alpha values typically ranging between 0.80 and 0.90, as well as satisfactory construct validity across different populations, including Arabic-speaking populations (Garner et al., 1982; Al-Subaie et al., 1996). Similarly, the Body Shape Questionnaire (BSQ-16B) has shown excellent internal consistency, with Cronbach’s alpha exceeding 0.90 in both the original and shortened versions (Evans and Dolan, 1993; Cooper et al., 1987). A pilot test was conducted prior to data collection to assess the validity, clarity, and cultural appropriateness of the Arabic versions of both instruments. The pilot sample consisted of approximately 10 undergraduate medical students who were representative of the target population but were not included in the final analysis. Participants were asked to complete the survey and provide feedback on the wording, ambiguity, and overall comprehensibility of the items. Based on the feedback received, minor linguistic modifications were made to improve clarity and ensure better understanding of the survey without altering the original meaning of the items. The pilot study confirmed that the questionnaire was well understood, appropriate for the target population, and did not require any major structural changes.

### Ethical considerations

Ethical approval was obtained from the University of Jordan Institutional Review Board (IRB) The study followed the guidelines of the Helsinki II Declaration, where participants provided informed consent after receiving a brief explanation of the study and its goals. They were also notified that their participation would be voluntary and that there would be no consequences from withdrawal.

### Data analysis

Data was analyzed using IBM SPSS Statistics (version 27). Descriptive statistics were used to summarize participant characteristics, with categorical variables presented as frequencies and percentages. Differences in EAT-26 risk categories (low vs. high risk) across participant characteristics were examined using independent-samples t tests for binary variables and one-way analysis of variance (ANOVA) for variables with more than two categories, with post-hoc tests performed where appropriate based on homogeneity of variances. Associations between categorical variables were assessed using the chi-square test. Body mass index (BMI) was categorized according to standard classifications. Participants with missing BMI data (*n* = 23) were excluded from analyses involving BMI.

Binary logistic regression using a forward stepwise likelihood ratio method was performed to identify factors independently associated with high EAT-26 risk. Model performance was evaluated using the Omnibus Tests of Model Coefficients, −2 log likelihood, Cox and Snell *R*^2^, and Nagelkerke *R*^2^, while model calibration was assessed using the Hosmer–Lemeshow goodness-of-fit test. Odds ratios (ORs) with 95% confidence intervals (CIs) were reported. A two-sided *p* value < 0.05 was considered statistically significant. The sequence of variable entry and stepwise model results are presented in Appendix A, Table A1. A two-sided *p* value < 0.05 was considered statistically significant.

## Results

The sociodemographic and clinical characteristics of the 402 participating students and their mean EAT-26 and BSQ-16B scores are presented in Table 1. Most participants were female (70.9%), aged between 18 and 20 years, had a normal BMI, and lived with their families. Differences in mean EAT-26 and BSQ-16B scores were observed across demographic and clinical subgroups.

**Table 1:** Sociodemographic characteristics and eating attitudes (EAT-26) and body shape (BSQ-16) scores of participants.

Item	Categories	N	%	Mean EAT-26 (SD)	*p*-value	Mean BSQ-16B (SD)	*p*-value
Gender	Female	285	70.90%	17.03 (12.91)	0.03*	47.24 (21.90)	0.95
	Male	117	29.10%	15.60 (12.10)		47.40 (21.38)	
Age	18–20	212	52.70%	16.80 (13.02)	0.26	45.77 (21.07)	0.24
	21–23	181	45.00%	16.41 (12.41)		48.75 (22.10)	
	24–26	5	1.70%	15.14 (12.00)		51.57 (32.31)	
	27 or more	4	0.50%	22.00 (2.82)		60.50 (7.78)	
BMI	Underweight	28	7.39%	14.04 (11.28)	0.014*	32.75 (17.94)	<0.001*
	Normal	226	59.63%	15.37(13.66)		41.76 (21.13)	
	Overweight	78	20.58%	18.53 (10.32)		56.30 (16.16)	
	Obese	47	12.40%	21.00 (11.65)		67.25 (15.77)	
University	Al-Balqa’ Applied University	28	7.00%	14.04 (12.93)	0.79	43.21 (21.95)	0.38
	Hashemite University	28	7.00%	15.86 (13.29)		40.61 (21.14)	
	Jordan University of Science and technology	19	4.70%	18.63 (12.08)		51.84 (16.59)	
	Mutah University	9	2.20%	17.33 (10.30)		50.44 (22.31)	
	University of Jordan	232	57.70%	17.14 (12.92)		47.63 (21.74)	
	Yarmouk University	86	21.40%	15.80 (12.22)		48.53 (22.68)	
Academic year	First	76	18.90%	17.33 (10.10)	0.9	52.84 (20.55)	0.07
	Second	108	26.90%	15.11 (11.97)		44.65 (22.09)	
	Third	131	32.60%	17.33 (10.10)		43.19 (21.56)	
	Fourth	54	13.40%	17.45 (11.70)		55.11 (28.06)	
	Fifth	24	6.00%	15.38 (12.68)		49.54 (22.02)	
	Sixth	9	2.20%	17.09 (13.57)		47.57 (21.36)	
GPA	Poor	4	1.00%	28.25 (13.77)	0.08	56.75 (21.23)	0.16
	Acceptable	13	3.20%	21.08 (12.87)		51.31 (26.05)	
	Good	76	18.90%	19.72 (14.23)		52.83 (22.73)	
	Very Good	161	40.00%	16.06 (11.94)		46.60 (20.43)	
	Excellent	148	36.80%	14.93 (12.20)		44.57 (21.85)	
Family income level per month (JOD)	Less than 500	31	7.70%	15.81 (10.96)	0.81	49.48 (19.74)	0.61
	500–999	87	21.60%	16.17 (13.84)		45.11 (22.98)	
	1,000–1,499	102	25.40%	16.44 (11.83)		45.65 (21.32)	
	1,500–2000	47	11.70%	15.45 (11.44)		49.21 (23.35)	
	More than 2000	135	33.60%	17.64 (13.37)		48.75 (21.13)	
Place of Residence	Rural	64	15.90%	14.42 (10.69)	0.13	43.75 (20.14)	0.16
	Urban	338	84.10%	17.04 (12.99)		47.96 (21.97)	
Who do you live with?	Alone	47	11.70%	19.93 (13.23)	0.16	54.66 (21.12)	0.03*
	In dorms	27	6.70%	18.98 (12.61)		49.63 (22.28)	
	With family	328	81.60%	16.01 (11.94)		46.00 (21.60)	
Governorate	Central Governorates (Amman, Zarqa, Balqa, Madaba)	286	71.10%	16.77 (12.44)	0.76	47.78 (22.11)	0.12
	Northern Governorates (Jerash, Ajloun, Irbid, Mafraq)	96	23.90%	15.88 (13.25)		46.78 (21.02)	
	Southern Governorates (Karak, Ma’an, Tafilah, Aqaba)	20	5.00%	18.05 (13.67)		42.60 (19.77)	
Marital status	Married	5	1.20%	26.00 (11.18)	0.13	62.60 (15.11)	0.08
	Single	397	98.80%	16.50 (12.66)		47.09 (21.74)	
Have you ever been diagnosed with a psychiatric illness?	No	375	93.30%	15.99(12.09)	0.047*	46.35(21.37)	0.03*
	Yes	27	6.70%	25.37(17.03)		60.22(22.90)	
Have you ever received psychiatric treatment?	No	376	93.50%	16.05(11.98)	0.08	46.38(21.27)	0.03*
	Yes	26	6.50%	24.85(18.66)		60.35(24.33)	
Have you used diet pills or laxatives to lose weight?	No	369	91.80%	15.52(11.87)	<0.001*	45.29(21.15)	<0.001*
	Yes	33	8.20%	28.97(14.95)		69.64(14.50)	
Have you lost more than 10 kg in 6 months?	No	281	69.90%	14.44(11.54)	<0.001*	43.06(21.35)	0.003*
	Yes	121	30.10%	21.69(13.76)		57.10(19.35)	
No. of Family Members	01-Mar	12	3.00%	20.50 (14.93)	0.6	56.08 (26.60)	0.45
	04-Jun	250	62.20%	16.09 (12.35)		47.45 (21.60)	
	6+	140	34.80%	16.74 (13.08)		46.24 (21.48)	
Has any family member been diagnosed with a mental health disorder?	No	358	89.10%	16.04(12.20)	0.1	46.03(21.33)	0.14
	Yes	44	10.90%	21.36(15.43)		57.55(22.41)	
Your position among siblings	Only child	5	1.20%	16.60 (13.22)	0.96	46.00 (20.22)	0.67
	Youngest	69	17.20%	17.41 (13.80)		50.03 (21.72)	
	Middle child	183	45.50%	16.38 (12.89)		46.25 (22.25)	
	Oldest	145	36.10%	16.55 (11.93)		47.14 (21.58)	

Subgroup analyses identified several significant associations. Female students had higher mean EAT-26 scores than males (*p* = 0.03), whereas BSQ-16B scores did not differ by gender. BMI was significantly associated with both outcomes. Post-hoc Tukey comparisons showed that obese participants had higher EAT-26 scores than those with normal BMI (*p* = 0.029), while no other BMI pairwise differences were significant. For BSQ-16B scores, Games-Howell post-hoc analysis revealed a graded increase across BMI categories, with overweight and obese participants reporting significantly greater body shape concerns than underweight and normal-weight individuals (all *p* < 0.001), and obese participants scoring higher than overweight participants (*p* = 0.002).

Who the participant lived with was also associated with BSQ-16B scores; Tukey post-hoc analysis indicated that students living alone were linked to higher body shape concern scores than those living with their families (*p* = 0.029), whereas no significant differences were observed for dormitory residence. Participants with a history of psychiatric illness were associated with higher mean EAT-26 and BSQ-16B scores (*p* = 0.047 and *p* = 0.03, respectively), and those who had received psychiatric treatment also had higher BSQ-16B scores (*p* = 0.03). In addition, participants who reported using diet pills or laxatives or who had lost more than 10 kg within 6 months demonstrated significantly higher scores on both measures (all *p* ≤ 0.003). No significant differences were observed across age groups, academic year, university, GPA, income level, place of residence, governorate, marital status, family size, sibling order, or family history of mental health disorders.

A Pearson correlation was performed to explore the relationship between EAT-26 and BSQ-16B scores. The results showed a strong, positive, and statistically significant correlation (*r* = 0.70, *p* < 0.001), suggesting that students with higher unhealthy eating attitudes tended to have greater concerns about their body shape.

Gender, BMI, psychiatric history, use of diet pills or laxatives, and recent significant weight loss were significantly associated with EAT-26 risk categories. Females were more likely to fall into the high-risk group compared to males (36.1% vs. 29.9%, *p* = 0.03). A clear gradient was observed across BMI categories, with a higher proportion of participants in the high-risk group as BMI increased, from 21.4% in underweight individuals to 54.7% in those with obesity (*p* = 0.006).

Participants with a history of psychiatric illness were more frequently classified as high risk (51.9% vs. 33.1%, *p* = 0.047). Furthermore, strong associations were seen in those who used diet pills or laxatives (72.7% high risk, *p* < 0.001) and those who had lost more than 10 kg in 6 months (50.4% high risk, *p* < 0.001). These associations are summarized in Table 2.

**Table 2:** Sociodemographic and clinical variables associated with EAT-26 risk categories (chi-square test).

Item	Categories	EAT-26				*p*-value
		Low risk		High risk		
		Count	%	Count	%	
Gender	F	182	63.9%	103	36.1%	0.03*
	M	82	70.1%	35	29.9%	
BMI	Underweight	22	78.6%	6	21.4%	<0.001*
	Normal	164	72.6%	59	27.4%	
	Overweight	43	55.1%	35	44.9%	
	Obese	21	44.7%	29	55.3%	
Have you ever been diagnosed with a psychiatric illness?	No	251	66.9%	124	33.1%	0.047*
	Yes	13	48.1%	14	51.9%	
Have you used diet pills or laxatives to lose weight?	No	255	69.1%	114	30.9%	<0.001*
	Yes	9	27.3%	24	72.7%	
Have you lost more than 10 kg in 6 months?	No	204	72.6%	77	27.4%	<0.001*
	Yes	60	49.6%	61	50.4%	

*Significant, *p* ≤ 0.05.

**Figure 1:**
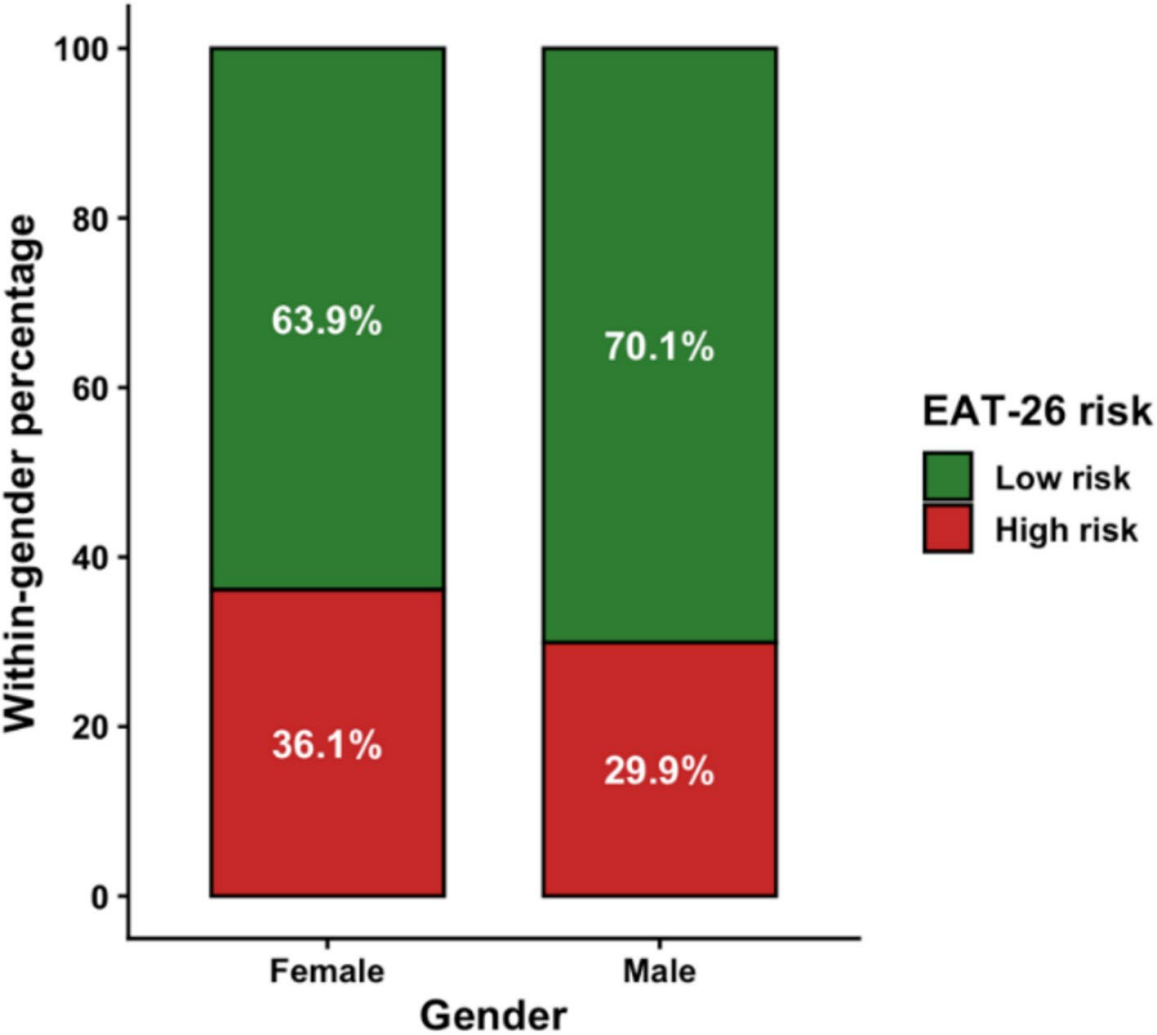
Distribution of EAT-26 risk categories by gender.

Figure 1 illustrates the within-gender distribution of eating-disorder risk as assessed by the Eating Attitudes Test-26 (EAT-26). A total of 138 participants (34.3%) fell under the high risk group. A higher proportion of females were classified as high risk compared with males (36.1% vs. 29.9%), while low-risk classifications were more common among males than females (70.1% vs. 63.9%). Values are expressed as within-gender percentages.

**Figure 2:**
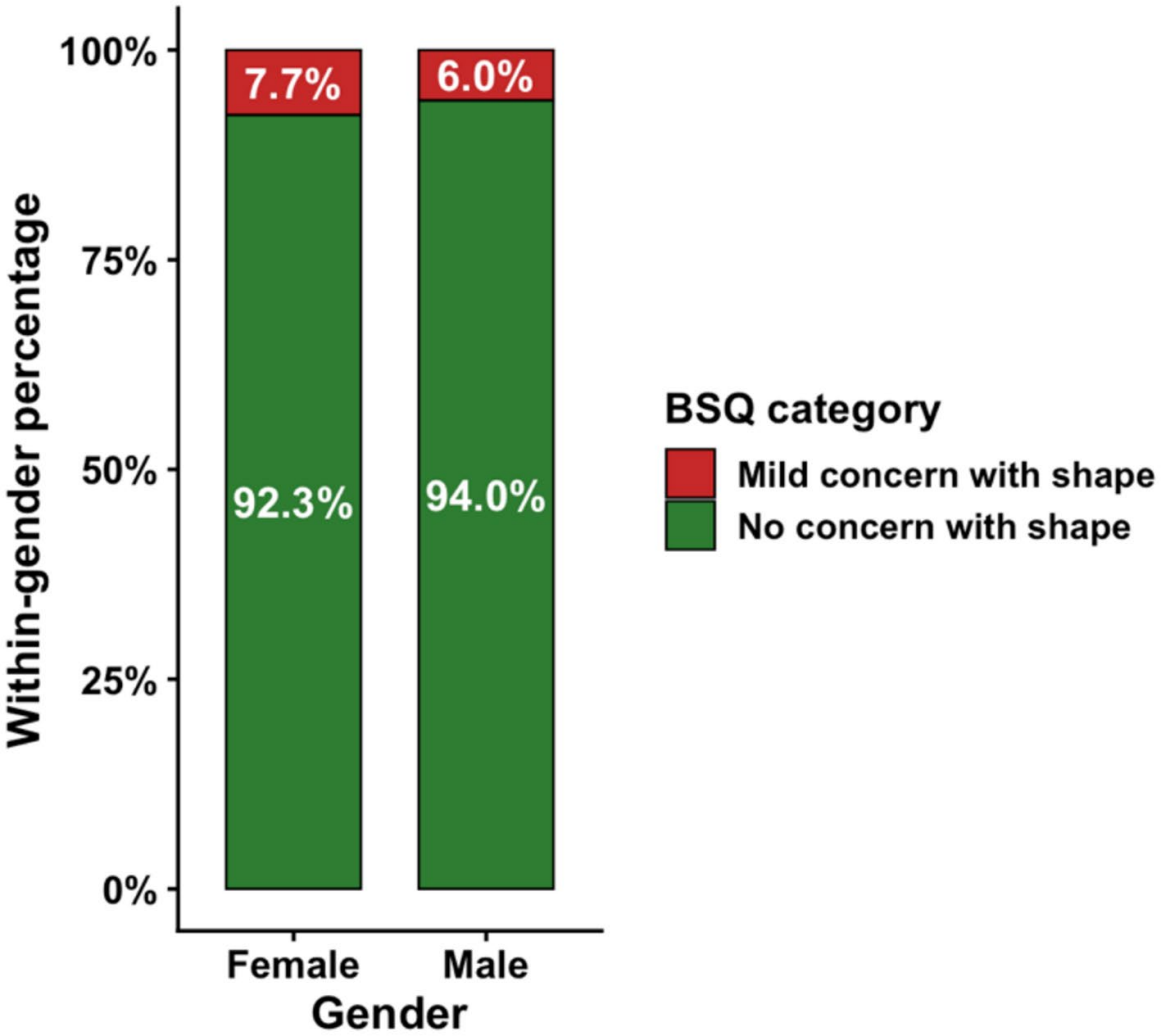
Distribution of BSQ-16 categories by gender.

Figure 2 demonstrates the within-gender distribute on of body shape concern as measured by the Body Shape Questionnaire (BSQ). A total of only 29 participants (7.2%) have mild concerns with their body shape. The majority of both genders reported no concern with shape, with males showing a slightly higher proportion than females (94.0% vs. 92.3%). Conversely, mild concern with shape was more prevalent among females compared with males (7.7% vs. 6.0%). Values are expressed as within-gender percentages.

A stepwise (forward likelihood-ratio) binary logistic regression analysis was conducted to identify factors independently associated with high eating-disorder risk. The overall model was statistically significant based on the Omnibus Likelihood Ratio test of model coefficients, *χ*^2^(6) = 51.1, *p* < 0.001, indicating that the included predictors significantly improved model fit compared with the null model. The model explained between 10.6 and 17.5% of the variance in eating-disorder risk (McFadden *R*^2^ = 0.106; Nagelkerke *R*^2^ = 0.175). Model calibration was adequate, as indicated by a non-significant Hosmer–Lemeshow test (*p* > 0.05). Multicollinearity was not a concern, with all predictors exhibiting low variance inflation factors (VIF < 1.1) and high tolerance values (> 0.9). Using a probability cut-off of 0.5, the model demonstrated higher specificity than sensitivity. It correctly classified 88.7% of participants in the low-risk group, whereas only 34.1% of high-risk participants were correctly identified. This indicates that the model was more effective at ruling out high eating-disorder risk than at detecting it, despite demonstrating acceptable overall discriminatory ability (AUC = 0.723).

Male gender was associated with significantly lower odds of being classified as high risk compared to females (OR = 0.54, 95% CI: 0.32–0.93; *p* = 0.026). In contrast, participants who reported using diet pills or laxatives demonstrated nearly fivefold higher odds of high-risk eating attitudes (OR = 4.95, 95% CI: 1.97–12.40; *p* < 0.001), while those who had lost more than 10 kg within the previous 6 months had more than twice the odds of being classified as high risk (OR = 2.10, 95% CI: 1.26–3.52; *p* = 0.005). Compared with underweight participants, individuals in the overweight (OR = 3.26, 95% CI: 1.12–9.51; *p* = 0.030) and obese (OR = 4.43, 95% CI: 1.42–13.84; *p* = 0.011) categories were significantly more likely to be in the high-risk group, whereas the difference between normal-weight and underweight participants was not statistically significant (*p* = 0.357). The results of the binary logistic regression analysis are presented in Table 3.

**Table 3:** Binary logistic regression analysis of factors associated with high EAT-26 risk.

Variable	Category	OR (95% CI)	*p*
Gender (Reference: Female)	Male	0.54 (0.32–0.93)	**0.026**
Have you used diet pills or laxatives to lose weight? (Reference: No)	Yes	4.95 (1.97–12.40)	< 0.001*
Have you lost more than 10 kg in 6 months? (Reference: No)	Yes	2.10 (1.26–3.52)	0.005*
BMI (Reference: Underweight)	Normal	1.60 (0.59–4.34)	0.357
	Overweight	3.26 (1.12–9.51)	0.030*
	Obese	4.43 (1.42–13.84)	0.011*

*Significant, *p* ≤ 0.05.

## Discussion

This study examined ED risk and body shape concerns among Jordanian medical students, finding that over one-third were classified as high-risk (screening-positive) based on EAT-26. Key associations included female gender, elevated BMI, psychiatric history, weight control medication use, and recent significant weight loss for ED risk, and BMI categories and living arrangements for body shape concerns. A strong positive correlation was also observed between eating attitudes and body shape dissatisfaction, which is consistent with the conceptual overlap between eating pathology and body image concerns, adding to growing evidence of disordered eating among medical students in the MENA region (Bizri et al., 2020; Dodin et al., 2024; Memon et al., 2012; Thomas et al., 2010; Alghamdi et al., 2018) and highlight specific risk factors warranting clinical attention and early intervention.

The proportion of participants classified as high-risk in Eat-26 (screening-positive) in this sample aligns with emerging patterns documented across medical education settings in the region. Medical students at Yarmouk University in Jordan demonstrated similar risk levels using alternative screening instruments (Dodin et al., 2024), suggesting consistency in the proportion of students classified as high-risk (screening-positive) across Jordanian medical institutions. Regional comparisons show comparable rates among medical students at King Abdulaziz University in Saudi Arabia (Ghamri et al., 2022), indicating that medical training settings across the Arab Gulf countries may share common stressors influencing eating pathology. The convergence of these data among different assessment tools and educational institutions strengthens the evidence that medical students encounter elevated vulnerability to disordered eating behaviors during their training period (Motorga et al., 2024). However, these findings should be interpreted with caution given the recruitment strategy. Online convenience sampling in eating disorder research is particularly susceptible to participation bias, as engagement with the survey may itself be shaped by body image concerns, personal interest in the topic, or conversely by stigma and avoidance among affected individuals. Our estimate of 34.3% reflects the proportion of participants screening-positive rather than confirmed clinical cases,and may over- or underestimate the true prevalence in the broader Jordanian medical student population.

Historical comparisons with earlier studies reveal a possibly increasing trend in ED risk among university populations in this region. Previous studies among female university students in the United Arab Emirates documented lower prevalence rates (Thomas et al., 2010), while studies at the American University of Beirut similarly reported lower frequencies (Bizri et al., 2020). The higher rates observed in contemporary cohorts may reflect evolving sociocultural pressures, increased academic demands, or improved detection through validated screening instruments (Ghamri et al., 2022; Dodin et al., 2024). Alternatively, the medical student population specifically may face unique pressures related to professional expectations, performance anxiety, and exposure to health-related information that distinguish them from general university samples (Motorga et al., 2024; Shaw et al., 2026). The substantial burden and demanding study time may push medical students to go for easily accessible unhealthy fast food and irregular eating patterns (AlJaber et al., 2019). Stress is associated with emotional eating, skipping meals, and favoring unhealthy comfort food which can lead to weight gain and malnutrition (Alzahrani et al., 2020).

The gender differential in ED risk observed in this study reflects well-established global patterns in which female students demonstrate higher frequencies of restrictive eating behaviors and weight control attempts (Tavolacci et al., 2015; Eisenberg et al., 2011). In Western societies beauty standards advocate for women thinness and low body fat (Stice and Shaw, 2002). On the contrary, beauty in Middle Eastern countries traditionally favored a plumper body, which was socially associated with fertility and wealth (Melisse et al., 2020; Pike and Dunne, 2015). With the growing globalization and rapid social media invasion of the Arab world, young women face confusion at the intersection between conventional beauty norms and modern trends. The dynamics of hybrid coexistence of culturally defined beauty criteria and prevailing global influences can lead to potential conflict between Eastern and Western beauty standards (Naji et al., 2025). The results of a cross-sectional study conducted in Qatar with 1,418 female university students using EAT-26, confirmed the association between social media use, unhealthy relationship with food and negative perception of body image (Qutteina et al., 2019).

Regression analyses showed that male gender was associated with lower odds of high-risk classification, consistent with sociocultural theories emphasizing differential pressure on women to conform to thin-ideal standards (Rothblum, 1992; Sattler et al., 2018). These gendered expectations appear especially evident in Middle Eastern contexts where cultural norms regarding female appearance remain influential (Thomas et al., 2010). However, the absence of gender differences in body shape concern scores adds an important complexity, suggesting that while females may engage more frequently in observable dieting behaviors, body dissatisfaction as an internal experience appears equally distributed across genders.

This difference between behavioral manifestations and subjective distress suggests that male medical students may experience body image concerns that express themselves through different pathways than those measured by traditional ED screening instruments (Montgomery Sklar, 2015). Emerging evidence from the Middle East indicates rising body image concerns among young men, potentially appearing as pursuit of muscularity rather than thinness (Ghazzawi et al., 2022). The comparable body dissatisfaction between genders despite divergent eating attitudes implies that male students may engage in alternative weight control behaviors not captured by the EAT-26, or that their concerns remain internalized rather than translated into restrictive eating patterns. This hidden burden of male body dissatisfaction warrants targeted investigation to understand its clinical significance and potential long-term consequences.

In our study, the likelihood of being screening-positive for ED risk increased progressively across BMI categories, supporting existing evidence that higher body weight is closely associated with body dissatisfaction and engagement in unhealthy weight-control practices. Participants with obesity demonstrated the highest risk across both measures, with stepwise increases in body shape concerns across ascending BMI categories. This likely reflects a bidirectional process whereby elevated weight intensifies body dissatisfaction, which in turn motivates maladaptive weight control behaviors that may paradoxically perpetuate further weight gain (Weinberger et al., 2016; Camacho-Barcia et al., 2024).

Weight stigma documented across Middle Eastern societies may amplify the psychological distress associated with elevated BMI beyond health-related concerns (Alghamdi et al., 2018). When the majority of students with obesity fall into the high-risk category rather than representing exceptional cases, this suggests that weight-related distress has become normative rather than exceptional in this weight group. Medical students with elevated BMI may face additional pressures related to perceived incongruence between their body size and professional expectations for physician appearance (Goldring and Persky, 2018), potentially intensifying their psychological burden beyond what general university students experience.

The association between diet pill or laxative use and ED risk is among the most clinically significant findings. Although few participants reported such use, those who did showed markedly elevated odds of high-risk status. In our sample, this pattern aligns with established evidence that pharmacological weight-control behaviors are strongly linked to disordered eating, suggesting that even infrequent use may signal underlying maladaptive coping strategies. This behavior frequently functions as a gateway to more severe eating pathology, with pharmacological weight control predicting progression to diagnosable eating disorders (Elsahory, 2021). The prevalence observed in this sample approximates rates documented among broader university populations, suggesting medical students do not face disproportionate risk for this specific behavior despite their enhanced access to pharmaceutical agents (Bizri et al., 2020; Tavolacci et al., 2015).

Medical students’ knowledge of pharmacology and potential access to prescription medications through clinical rotations may facilitate self-medication behaviors that other university students would find more difficult to initiate (Arora et al., 2016). The combination of academic pressure, body dissatisfaction, and pharmaceutical knowledge may represent a unique vulnerability specific to medical training environments. Students engaging in pharmacological weight control likely represent a subgroup experiencing significant distress whose behaviors may signal a need for further clinical evaluation rather than simple health education. The presence of this behavior pattern should prompt comprehensive assessment for underlying eating pathology rather than being dismissed as misguided dieting attempts.

Recent significant weight loss was independently associated with higher ED risk, with regression analyses demonstrating that participants reporting loss of more than 10 kg in 6 months faced substantially elevated odds of risk classification. In our cohort, individuals reporting recent substantial weight loss were more likely to be classified as screening-positive for ED risk, which is consistent with prior literature linking rapid weight change to restrictive or compensatory eating behaviors. While intentional weight loss may sometimes reflect appropriate health behavior, the magnitude and timeframe give rise to concerns about potentially dangerous restriction patterns (Habib et al., 2023). Rapid weight loss in medical students may reflect extreme dieting practices, excessive exercise, or combination approaches that interfere with academic performance and physical health. The clustering of this variable with other associated factors in regression models suggests it identifies students whose weight control efforts have escalated beyond normative dieting into pathological territory.

The differentiation between intentional health-focused weight management and disordered eating-driven weight loss remains clinically challenging without detailed behavioral assessment (Breda et al., 2024; Withnell and Bodell, 2025). However, the strong statistical association between recent large-magnitude weight loss and high ED risk indicates that this easily identifiable marker may warrent clinical attention and further evaluation.

Living arrangements demonstrated specific associations with body shape concerns, with students residing alone reporting higher distress than those living with families. This finding suggests that family presence may provide protective buffering against body image preoccupation, potentially through regular structured meals, reduced opportunity for secretive eating behaviors, or emotional support that mitigate appearance-related distress (Lavell et al., 2025; Izydorczyk et al., 2021). Jordanian culture emphasizes collectivist values and strong family ties, with family structures traditionally providing psychological support during stressful life transitions including university education (Al-Hassan, 2024).

Students living alone may lack this protective scaffolding while simultaneously facing increased autonomy regarding food choices and eating patterns. Evidence from neighboring countries indicates that students in independent living situations demonstrate higher prevalence of irregular eating habits and meal skipping compared to those living with families (Mikhael et al., 2018). The isolation associated with living alone may also amplify self-focused attention on appearance and body evaluation without the normalizing influence of family perspectives (Barnett et al., 2020). However, the absence of significant associations between living arrangement and eating attitudes suggests this protective effect may be specific to subjective body image concerns rather than broadly linked to eating behaviors.

Psychiatric history demonstrated expected associations with both ED risk and body shape concerns, with substantial proportions of participants with prior mental health conditions falling into high-risk categories. This finding is in line with well-established comorbidity patterns in which ED frequently co-occur with mood disorders, anxiety conditions, and other psychiatric diagnoses (Hambleton et al., 2022). Students with psychiatric histories may possess underlying vulnerabilities including emotion regulation difficulties, perfectionistic traits, or neural reward processing differences that increase susceptibility to eating pathology when confronted with medical training stressors.

The directional relationship between psychiatric conditions and ED development is still difficult to establish through cross-sectional data, as these associations likely reflect complex bidirectional influences (Hambleton et al., 2022). ED may emerge as coping mechanisms in students struggling with pre-existing anxiety or depression, while alternatively, chronic eating pathology may precipitate mood disturbances through nutritional deficiencies and psychological distress (Strete et al., 2025).

Contrary to hypotheses that academic progression influences ED risk, this study found no significant associations with academic year, grade point average, or institution attended. These findings contest assumptions that eating pathology peaks during specific high-stress periods such as pre-clinical examination years or clinical rotation initiation. Instead, the results suggest that ED risk remains elevated throughout medical training rather than concentrating in phases. This pattern in line with multinational investigations in the Middle East documenting complex non-linear relationships between academic stage and eating pathology (Belhaj Salem et al., 2025), contrasting with some Saudi Arabian studies identifying heightened risk in specific training years (Ghamri et al., 2022).

The absence of academic variable associations implies that ED vulnerability in medical students may primarily reflect individual psychological characteristics and general medical school environmental factors rather than responses to curricular demands or performance pressure. Students predisposed to eating pathology through personality traits, family history, or prior weight concerns may develop disorders at any point during training when environmental triggers combine with underlying vulnerabilities (Barakat et al., 2022). This suggests that prevention and intervention efforts have to maintain consistent availability throughout all training years rather than concentrating resources on presumed high-risk periods.

Similarly, the lack of association between grade point average and ED risk indicates that academic achievement does not systematically relate to eating pathology in this population. High-achieving students do not appear protected from ED, nor do struggling students demonstrate elevated risk. This contrasts with perfectionism research linking extremely high achievement orientation with ED vulnerability (Sundquist et al., 2015), though perfectionism may manifest through different pathways than observable academic performance.

The robust positive correlation between eating attitudes and body shape concerns documented in this study reflects the theoretically predicted relationship where cognitive and behavioral eating pathology co-occurs with perceptual and affective body image disturbance. Students exhibiting restrictive eating attitudes typically harbor significant body dissatisfaction, with the two often co-occurring. This strong correlation validates the conceptual framework linking cognitive distortions about eating with negative body image as interconnected components of ED psychopathology (Zhao et al., 2024). The relationship suggests that interventions targeting either eating attitudes or body image concerns alone may provide incomplete treatment, with comprehensive approaches addressing both domains potentially achieving superior outcomes.

However, the correlation also shows that these constructions remain partially independent despite their strong relationship, as evidenced by individuals scoring high on one measure but not the other. Some students may experience significant body dissatisfaction without translating these concerns into restrictive eating behaviors, potentially engaging in alternative coping mechanisms or lacking specific weight control knowledge. Conversely, some students may adopt restrictive eating patterns motivated by factors beyond appearance concerns, such as gastrointestinal issues, athletic performance goals, or religious fasting practices that exceed normative observance (Sundquist et al., 2015). Understanding these different presentations requires clinical assessment beyond screening instruments to clarify underlying motivations and maintaining factors.

Taken together, the identified associations should be interpreted as part of a more complex picture rather than a complete explanation of ED risk in this population. The regression model explained only a modest proportion of the variance in high-risk classification and showed limited sensitivity, indicating that the demographic and behavioral variables examined here capture only some of the relevant associated factors. Additional psychological and contextual factors, including perfectionism, emotion regulation, interpersonal difficulties, trauma history, peer and family dynamics, and broader sociocultural exposures, are likely involved and warrant further investigation in future research.

### Limitations

The reliance on self-report instruments introduces potential measurement limitations related to social desirability bias, recall accuracy, and varying interpretation of the questionnaire items. Students may underreport stigmatized behaviors such as purging or diet pill use, leading to underestimation of their true prevalence. The EAT-26 identifies the risk for ED rather than providing diagnostic confirmation, with elevated scores indicating the need for further clinical evaluation rather than establishing disease presence. Some students scoring above risk thresholds may reflect normative dieting practices in weight-conscious populations rather than pathological eating patterns. Conversely, some students with emerging ED may score below cutoffs, if their behaviors do not align with the questionnaire items or if denial minimizes the symptoms’ acknowledgment. The cross-sectional design employed in this study precludes determination of temporal relationships between the identified associated factors and ED development. Elevated BMI may precede and contribute to eating pathological development or alternatively may result from cycles of restrictive eating and compensatory behaviors. Similarly, psychiatric conditions may increase ED vulnerability or may emerge secondary to chronic eating pathology and its physiological consequences.

Convenience sampling via students’ social media groups and online platforms carries the risk of selection bias. This is a significant limitation of this study because students with eating disorders or body image concerns might be either reluctant or more likely to respond to the online questionnaire which could lead to non-random participation bias and limit the generalizability to other contexts.

Females comprised a large majority of participants in this study which reflected the gender demographics in the Jordanian medical education. While this distribution mirrors the medical students’ population from which the sample was drawn, the relatively small male subsample constrains statistical power for gender-specific analyses and may obscure male-specific risk patterns. Relatedly, while the sample size was adequate for the primary aim of estimating the proportion of participants at elevated eating disorder risk, the study was not specifically powered for all subgroup comparisons or for the full multivariable models. Findings from less represented subgroups and the analyses involving multiple covariates should be interpreted with appropriate caution, particularly where confidence intervals are wide or effect estimates are based on cells with small frequencies.

The nonprobability sampling approach and single-country focus raise questions about whether the findings can be applied to other Middle Eastern nations with different cultural contexts, or to medical students in Western countries with alternative training structures and sociocultural pressures. Replication studies throughout diverse medical education settings would establish the cross-cultural consistency of identified risk patterns.

The regression model developed in this study demonstrated acceptable discriminatory ability but revealed differential performance in identifying high-risk versus low-risk students. The model reliably ruled out ED risk with high specificity, correctly classifying the majority of students without problematic eating attitudes. However, sensitivity remained limited, correctly identifying only a minority of high-risk students. This pattern suggests that easily measurable demographic and behavioral variables capture some but not all determinants of ED vulnerability. Psychological factors including perfectionism, emotion regulation difficulties, interpersonal problems, and trauma history likely contribute to additional unexplained variance. A further limitation concerns our use of stepwise variable selection, which can yield unstable models, inflate effect sizes, and underestimate standard errors, particularly in datasets with correlated predictors. Findings from the multivariable analysis should therefore be interpreted as exploratory rather than definitive and require confirmation in larger samples using pre-specified variable selection or regularization approaches.

### Strengths

The dual-instrument approach assessing both eating attitudes and body shape concerns, allowing differentiation between behavioral symptoms and affective body image components revealed the hidden burden of male body dissatisfaction that might have been missed through eating attitude assessment alone. The focus on medical students provides actionable data for a specific high-risk population where directed interventions could be implemented through existing student health infrastructure. The use of validated screening instruments with established psychometric properties in similar populations strengthens confidence in the measurement quality. The sample size provided adequate power for detecting associations with moderate to large effect sizes, though smaller effects may have been missed.

### Implications for practice

These findings areas warranting further research and targeted preventive strategies within medical education. Future research should examine whether universal ED screening could be feasibly incorporated into routine student health assessments. Students living alone may benefit from enhanced support structures such as peer networks or wellness check-ins.

Curricula should address weight stigma, healthy weight management, and ED warning signs both to protect students and to improve their future clinical practice. Institutional cultures that normalize help-seeking and challenge appearance-based discrimination would further reduce barriers to treatment.

Future research should employ longitudinal designs to establish temporal relationships between risk factors and ED development, with particular attention to critical transition points such as the pre-clinical to clinical shift. Studies using muscularity-focused instruments would better capture male body image pathology, while qualitative research could further inform intervention design and uncover risk factors not captured quantitatively.

## Conclusion

This study focused on ED risk among medical students in Jordan and revealed that a considerable proportion were screening-positive for elevated ED risk. Also, various associated factors were found to be significantly associated with elevated ED risk, with different correlations, including female students, those with a higher BMI, rapid weight loss, use of diet pills or laxatives, psychiatric history, and greater body shape concerns. Also, a strong correlation between ED and BID was noted, consistent with the established relationship between disordered eating and body image concerns. These findings are important because eating disorders are often unrecognized psychological conditions that can significantly affect adolescents and young adults, particularly medical students given the academic and psychological pressure they live through. However, this study had some limitations, including reliance on self-reported data, possible response bias, unequal gender distribution, and limited generalizability as the study was conducted in one country. Raising awareness may help prevent these problems, such as holding discussions on campus, listening to students’ concerns, early detection through screening, and student support programs that help them modify their lifestyle habits and rebuild a healthy relationship with food. Furthermore, more research is needed to explore ways to prevent these disorders and to develop effective intervention strategies that support both the mental and physical health of medical students.

## Data Availability

The raw data supporting the conclusions of this article will be made available by the authors, without undue reservation.
