# Supplementary material for: Eating disorders risk and body shape concerns among medical students in Jordan: a cross-sectional study
Source: Front Psychol. 2026 Jun 3;17:1816029. doi: 10.3389/fpsyg.2026.1816029 (PMC13272038; doi:10.3389/fpsyg.2026.1816029)
Supplement: Supplementary file 1 [file Supplementary_file_1.DOCX]

**Appendix A**

A stepwise forward binary logistic regression model was used to examine factors associated with high EAT-26 risk. The Omnibus Tests of Model Coefficients indicated that the model was statistically significant at each step, with progressive improvements in model fit as predictors were added. The final model (Step 4) was significant (χ²(6) = 51.15, p < 0.001), demonstrating a meaningful improvement over the null model. Model explanatory power increased across steps, with Nagelkerke R² rising from 0.075 in Step 1 to 0.175 in the final model, indicating a modest but meaningful proportion of variance explained. The Hosmer–Lemeshow goodness-of-fit test was non-significant in the final step (χ² = 3.44, df = 6, p = 0.752), suggesting adequate model calibration. As detailed in Appendix Table A1, the final model identified male gender as a protective factor, while the use of diet pills or laxatives, significant recent weight loss, and higher BMI categories were independently associated with increased odds of high EAT-26 risk

Table A1. **Logistic regression model diagnostics and stepwise performance**

|  | Variable | Category | Wald | Sig. | Exp(B) |
| --- | --- | --- | --- | --- | --- |
| Step 1a | **Have you used diet pills or laxatives to lose weight?** (Reference: No) | Yes | 17.73 | 0.001 | 6.694 |
| Step 2b | **Have you used diet pills or laxatives to lose weight?** (Reference: No) | Yes | 13.232 | 0.001 | 5.369 |
|  | **Have you lost more than 10 kg in 6 months?** (Reference: No) | Yes | 13.911 | 0.001 | 2.455 |
| Step 3c | **Have you used diet pills or laxatives to lose weight?** (Reference: No) | Yes | 12.875 | 0.001 | 5.335 |
|  | **Have you lost more than 10 kg in 6 months?** (Reference: No) | Yes | 6.411 | 0.011 | 1.913 |
|  | **BMI** (Reference: Underweight) | Normal | 0.458 | 0.498 | 1.408 |
|  |  | Overweight | 3.426 | 0.064 | 2.706 |
|  |  | Obese | 4.737 | 0.03 | 3.44 |
| Step 4d | Gender (Reference: Female) | Male | 4.959 | 0.026 | 0.542 |
|  | **Have you used diet pills or laxatives to lose weight?** (Reference: No) | Yes | 11.607 | 0.001 | 4.95 |
|  | **Have you lost more than 10 kg in 6 months?** (Reference: No) | Yes | 7.979 | 0.005 | 2.1 |
|  | **BMI** (Reference: Underweight) | Normal | 0.847 | 0.357 | 1.598 |
|  |  | Overweight | 4.689 | 0.03 | 3.259 |
|  |  | Obese | 6.544 | 0.011 | 4.425 |

| a Variable(s) entered on step 1: Have you used diet pills or laxatives to lose weight?. |
| --- |
| b Variable(s) entered on step 2: Have you lost more than 10 kg in 6 months?. |
| c Variable(s) entered on step 3: BMI. |
| d Variable(s) entered on step 4: Gender. |
